# Non-heparinized ECMO serves a rescue method in a multitrauma patient combining pulmonary contusion and nonoperative internal bleeding: a case report and literature review

**DOI:** 10.1186/s13017-015-0006-9

**Published:** 2015-03-12

**Authors:** Pei-Hung Wen, Wai Hung Chan, Ying-Cheng Chen, Yao-Li Chen, Chien-Pin Chan, Ping-Yi Lin

**Affiliations:** General Surgery Division, Surgery Department, Changhua Christian Hospital, Changhua City, Taiwan; Trauma Division, Surgery Department, Changhua Christian Hospital, 500 No. 135, Nanxiao Street, Changhua City, Taiwan; Cardiovascular Division, Surgery Department, Changhua Christian Hospital, Changhua City, Taiwan; Transplant Medicine and Surgery Research Centre, Changhua Christian Hospital, Changhua City, Taiwan; Surgery Department, Cishan Hospital, 84247 No. 60, Zhongxue Rd., Cishan District, Kaohsiung City, Taiwan

**Keywords:** ECMO, Heparin-free, Polytraumatic, Acute pulmonary contusion, Internal bleeding

## Abstract

Pulmonary contusion and acute respiratory distress syndrome (ARDS) is a common manifestation in polytraumatic patients. Although mechanical ventilation is still the first choice of treatment, a group of patients are still unable to maintain their oxygenation. The role of extracorporeal membrane oxygenation (ECMO) has been more clarified when the lung is extensively damaged and when conventional modality failed. ECMO provides the lung an opportunity to rest by permitting reduced ventilator settings and limiting further barotraumas. However, ECMO is still considered contraindicated in polytramatic patients combining pulmonary contusion and other organ hemorrhage because of systemic anticoagulation during the treatment. We herein report a patient who successfully survive a multitrauma combining pulmonary contusion and grade IV liver laceration using non-heparinized venovenous extracorporeal membrane oxygenation (vv-ECMO). The associated literature were reviewed.

## Introduction

Acute pulmonary failure is a common manifestation in polytraumatic patients. The mechanism included pulmonary contusion, acute respiratory distress syndrome (ARDS) resulting from any inflammation process such as aspiration pneumonia, or fat embolism because of long bone fracture [1]. The management could be very challenging despite advances in critical care management.

Extracorporeal membrane oxygenation (ECMO) serves as the final method when conventional mechanical ventilation fails to maintain the oxygenation. It helps maintain systemic tissue oxygenation via extracorporeal circuit when the lung function is compromised [2,3]. However, the situation became more complicated when the lung failure combines other vital organ damage and risk of bleeding because of systemic anticoagulation during the treatment.

The introduction of heparin-free ECMO seems to be the possible solution for such dilemma. We report our experience of using heparin-free vv-ECMO to help a patient survive a trauma combining acute pulmonary failure and severe liver subcapsular laceration. The literature regarding the application of ECMO in polytraumatic patients combining acute pulmonary failure and other vital organ damage were reviewed.

## Case report

**Figure 1:**
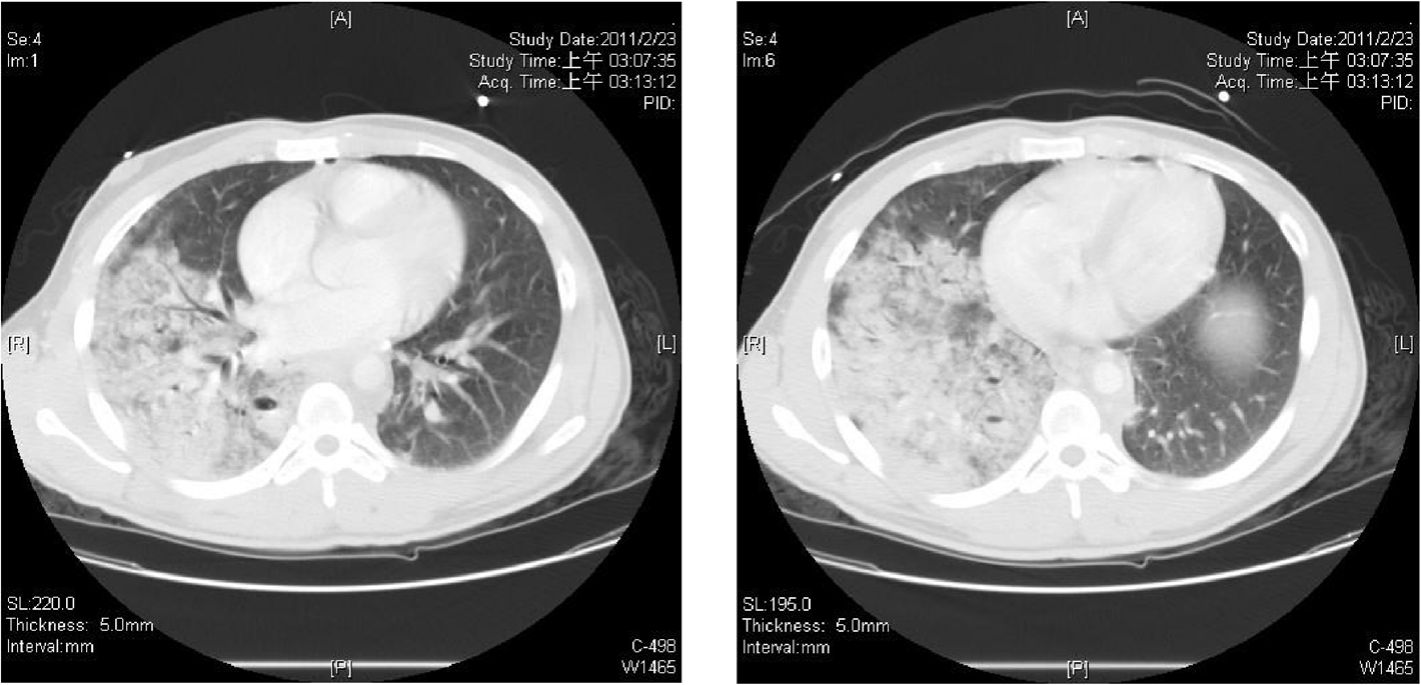
**Chest CT showed right lung consolidation with patchy opacities.**

**Figure 2:**
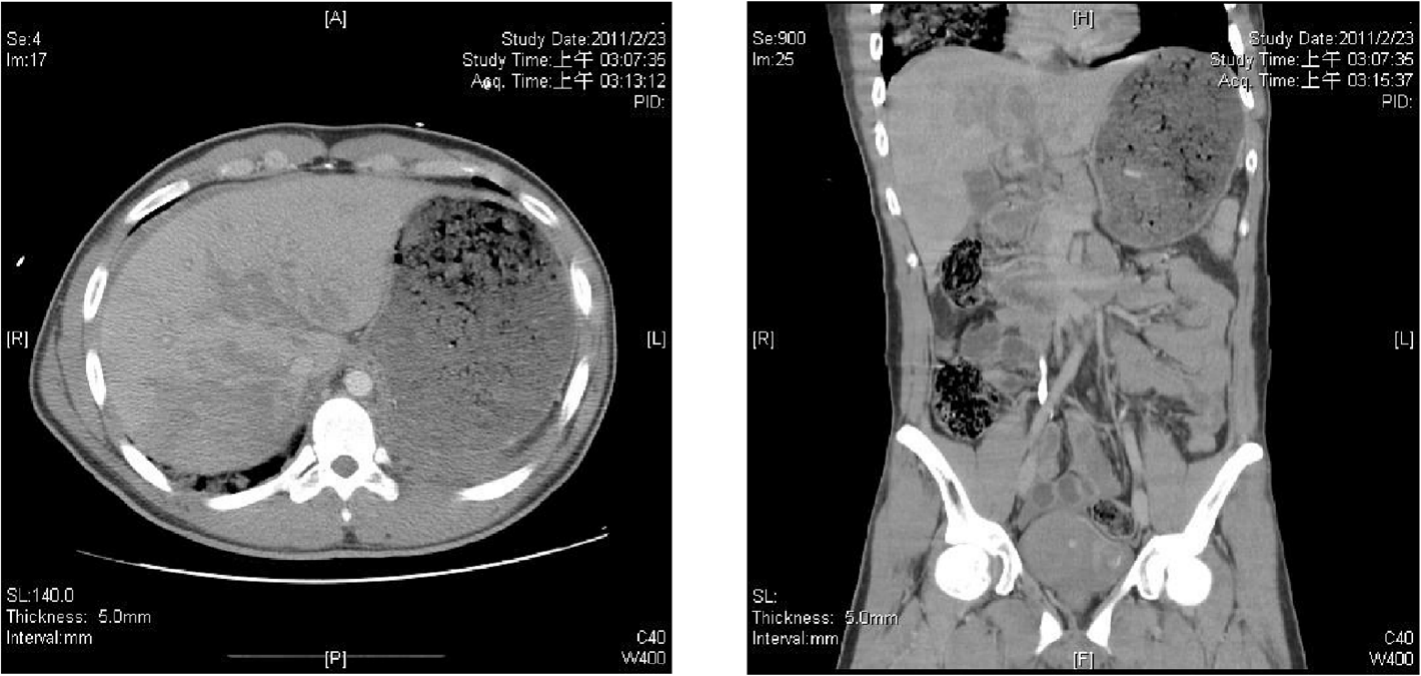
**The coronal view and saggital view of abdominal CT showed grade IV laceration over bilateral hemiliver without evident contrast extravasation.**

A 19-year-old man suffered from a multi-trauma after a traffic accident when he was riding a motorcycle and collided into a car. Upon arrival at our tertiary trauma center, he initially presented with a Glasgow Coma Score of 8 and severe hypoxia. He was intubated immediately. Large amount of food content was sucked from the endotracheal tube. Primary chest computed tomography (CT) reported right lung consolidation with patchy opacities, which was consistent with a combination of blunt chest contusion and aspiration pneumonia (Figure 1). Abdominal CT showed grade IV laceration over bilateral hemiliver without evident contrast extravasation (Figure 2). The gas exchange did not improve despite of mechanical ventilation, and the patient still presented with severe hypoxemia. The parameter about respiration showed PaO2/FiO2: 70.2 under invasive ventilation (pressure-mode inverse ratio ventilation, I:E 2:1). ECMO was thus recommended for lung contusion, aspiration pneumonia and acute pulmonary failure.

With regards of a polytraumatic patient combining liver laceration, ECMO is contraindicated because the need of systemic anticoagulation may induce further internal bleeding. A heparin-free, vv-ECMO was thus suggested. An extracorporeal circuit was constructed via a venous access through internal jugular vein and femoral vein, using Seldinger technique. The blood flow was set at the rate of 2.42 L/min, and the FiO2 was set at 45%. The oxygenation status improved dramatically after the introduction of ECMO (Table 1). The liver laceration was treated conservatively. The patient weaned from ECMO five days later, and was extubated nine days later. The total intensive care unit stay was 10 days, and he discharged after a sixteen-day hospitalization. There was no ECMO related complications during the course.

**Table 1:** **Oxygenation status before and after ECMO introduction**

	**Pre-ECMO**	**Post-ECMO**	**Pre-weaning**
pH	7.231	7.352	7.440
pCO2 (mmHg)	58.4	39.2	30.4
pO2 (mmHg)	70.2	147.2	91.5
O2 Saturation (%)	89.5	98.5	97.9

## Literature review

We searched the PubMed (2000–2013) database for case reports about the launch of ECMO regardless of heparin-containing or heparin-free in multi-trauma patients. The abstracts of all articles published in English were screened. The full texts of articles published in other languages but with an abstract in English were analyzed. Articles were selected for review if they included the following patient data: age, sex, clinical presentation, combined injury besides acute pulmonary failure, the details of ECMO treatment, and the outcome.

There were six case reports containing 11 patients described in detail, and one clinical paper containing 10 cases found in the literature, which are listed in Table 2.

**Table 2:** **ECMO in polytraumatic patients combining acute pulmonary failure and other vital organ damage: literature review**

**References**	**Case no.**	**Combined injury besides pulmonary failure**	**Intervention**	**ECMO**	**Heparin**	**ECMO duration**	**Outcome**
Madershahian et al. [2]	1, 19/F	Spleen, Liver	Laparotomy	v-a^5^	(+)	138 hours	Survived
		Right main bronchus	Thoracotomy				
	2, 48/M	Vertebra and long bone Fracture	Osteosynthesis	v-a	(+)	120 hours	Survived
	3, 26/M	Spleen	Splenectomy	v-va^6^	(+)	84 hours	Survived
		Brain					
Yuan et al. [5]	4, 18/M	Liver, Gr. III	Conservative	v-v	(+)	10 days	Survived
		Endobronchial hemorrhage					
	5, 38/M	Brain SDH^1^	Conservative	v-v	(+)	5 days	Survived
Campione et al. [4]	6, 14/M	Bronchial Disruption	Right bilobectomy of lung	v-v	(+)	3 days	Survived
Yen et al. [7]	7, 21/M	Brain EDH^2^	Decompressive craniotomy	v-a	(+)	49 hours	Survived
Friesenecker, et al. [8]	8, 34/M	Liver, Spleen	Laparotomy	v-v	(+)	17 days	Survived
		Brain ICH^3^ with edema	Decompressive craniotomy				
Muellenbach et al. [9]	9, 53/M	Liver	Laparotomy	v-v	(−)	8 days	Survived
		Traumatic brain injury	ICP^4^ Monitoring				
	10, 16/M	Traumatic brain injury		v-v	(−)	3 days	Survived
	11, 28/M	Spleen	Splenectomy	v-v	(−)	2 days	Survived
		Traumatic brain injury					
Arlt et al. [6]	10 Cases	Bleeding shock	-	7 v-v	All (−)	Mean 5 days	6/10 Survived
				3 v-a			

^1^SDH: Subdural hemorrhage; ^2^EDH: Epidural hemorrhage; ^3^ICH: Intracerebral hemorrhage; ^4^ICP: Intracerebral pressure; ^5^V-a: Venoarterial ; ^6^V-va: veno-venoarterial.

## Discussion

Despite of the various mechanical ventilation technique and the improved knowledge of the adjustment of ventilation parameters, a group of patients with traumatic pulmonary contusion or ARDS are still unable to benefit from these technique. ECMO has been proved to be an rescue therapy when conventional methods are ineffective. ECMO was also reported to be effective in polytraumatic patients combining pulmonary contusion and other organ damage including bronchial rupture [2,4], endobronchial hemorrhage [5], blunt abdominal trauma (BAT) with internal bleeding necessitating exploratory laparotomy [6], or traumatic brain injury [7-10]. However, the use of ECMO on patients with a preexisting bleeding risk without need of immediate operation is still rarely reported.

The application of heparin-free ECMO has been proposed recently to overcome the dilemma. Muellenbach et al. reported three successful cases of heparin-free vv-ECMO on a patient with traumatic lung failure and severe traumatic brain injury [9]. Matthias et al. reported that six of ten polytraumatic patients with coexisting pulmonary failure or cardiopulmonary failure and bleeding shock survived using a heparin-free ECMO, which is by far the only largest series in the literature [6]. However, ECMO is still a controversy on a multitrauma combining pulmonary failure and blunt abdominal trauma needing only nonoperative management. Of the total 11 cases reported in detail in the literature (Table 2), six patients had concurrent BAT with liver or spleen laceration [2,4,8,9]. Only one received successful nonoperative treatment for grade III liver laceration [4].

The improvement of ECMO technique including centrifugal pumps and heparin-coated circuits reduced the amount of heparin needed. However, with regards to a patient who had spontaneous hemostasis on liver laceration, we still chose the heparin-free method to reduce the risk of rebleeding. Based on the hemodynamic stability and the daily improvement of lung condition, we did not used additional method to prevent clotting of the circuit except the close monitor of ACT. The duration of ECMO was five days, which is comparable to other report.

## Conclusion

ECMO can serve as a rescue method to provide the traumatic lung to rest. Although it was previously regarded to be contraindicated in polytraumatic patient with coexisting organ hemorrhage, there are growing successful experiences reported recently. We report a heparin-free, vv-ECMO method for patients combining acute pulmonary failure and nonoperative liver laceration, which may extend the feasibility of ECMO in polytraumatic patients.

### Consent

Written informed consent was obtained from the patient for the publication of this report and any accompanying images.

## Abbreviations

ECMO: Extracorporeal membrane oxygenation
ARDS: Acute respiratory distress syndrome
v-v ECMO: Venovenous ECMO
CT: Computed tomography
FiO_2_: Fraction of inspired oxygen
BAT: Blunt abdominal trauma
SDH: Subdural hemorrhage
EDH: Epidural hemorrhage
ICH: Intracerebral hemorrhage
ICP: Intracerebral pressure
V-a ECMO: Venoarterial ECMO
V-va ECMO: Veno-venoarterial ECMO
